# Is restoration of vertebral body height after vertebral body fractures and minimally-invasive dorsal stabilization with polyaxial pedicle screws just an illusion?

**DOI:** 10.1007/s00402-023-05082-8

**Published:** 2023-10-15

**Authors:** Simon Thelen, Lisa Oezel, Lena Hilss, Jan-Peter Grassmann, Marcel Betsch, Michael Wild

**Affiliations:** 1 Department of Orthopaedics and Trauma Surgery, University Hospital Duesseldorf, Moorenstraße 5, 40225 Duesseldorf, Germany; 2 Department of Orthopaedics, Trauma- and Hand Surgery, Klinikum Darmstadt, Darmstadt, Germany; 3 Department of Orthopaedics and Trauma Surgery, University Hospital Erlangen, Erlangen, Germany

**Keywords:** Thoracolumbar fractures, Minimal invasive posterior stabilization, Polyaxial pedicle screws, Kyphotic deformity, Sagittal alignment

## Abstract

**Introduction:**

Thoracolumbar spine fractures often require surgical treatment as they are associated with spinal instability. Optimal operative techniques and treatment are discussed controversially. Aim of our prospective cohort study was to investigate the sagittal alignment after reduction, the secondary loss of reduction and the subjective outcome as well as the causal correlation of these parameters after minimally invasive stabilization of thoracic and lumbar fractures with polyaxial pedicle screws.

**Materials and methods:**

In a single-center study, a total of 78 patients with an average age of 61 ± 17 years who suffered a fracture of the thoracic or lumbar spine were included and subjected to a clinical and radiological follow-up examination after 8.5 ± 8 months. The kyphotic deformity was measured by determining the vertebral body angle, the mono- and bi-segmental wedge angle at three time points. The patients’ subjective outcome was evaluated by the VAS spine score.

**Results:**

After surgical therapy, a significant reduction of the traumatic kyphotic deformity was shown with an improvement of all angles (vertebral body angle: 3.2° ± 4.4°, mono- and bi-segmental wedge angle: 3.1° ± 5.6°, 2.0° ± 6.3°). After follow-up, a significant loss of sagittal alignment was observed for all measured parameters with a loss of correction. However, no correlation between the loss of reduction and the subjective outcome regarding the VAS spine scale could be detected.

**Conclusion:**

The minimally invasive dorsal stabilization of thoracic and lumbar spine fractures with polyaxial pedicle screws achieved a satisfactory reduction of the fracture-induced kyphotic deformity immediately postoperatively with a floss of reduction in the further course. However, maybe the main goal of this surgical procedure should be the prevention of a complete collapse of the vertebral body instead of a long-lasting restoration of anatomic sagittal alignment.

**Level of evidence:**

II.

**Supplementary Information:**

The online version contains supplementary material available at 10.1007/s00402-023-05082-8.

## Introduction

In recent years, an increase of spinal injuries has been observed, especially ascribed to demographic changes with increasing levels of mobility and activity in old age. In parallel, the number of spinal surgeries increased as well leading to social and socio-economic implications [1]. In younger patients, the most common causes of these injuries are high-energy traumas such as falls from great heights, traffic accidents, sports- and work-related accidents. This is in contrast to older patients, where low-energy falls at home are often the cause for spinal injuries [2–6]. Most spine fractures affect the thoracic and lumbar spine, with 65–70% of fractures located at the thoracolumbar junction [3–5]. The increased fracture risk in this area can be explained biomechanically by the fact that the thoracic spine is more rigid due to its costal attachments, while the lumbar spine is more flexible [2, 4, 5].

Treatment goals of spine injuries include the achievement of spinal stability, prevention of spinal cord injuries, and the restoration of spinal alignment in the sagittal and frontal planes, for which a large number of therapeutic options are available [3–5]. In recent years, dorsal, minimally invasive stabilization of spinal fractures using polyaxial pedicle screws has been well established [1, 4, 6–8]. This procedure can correct the kyphotic deformity caused by the fracture and restores the biomechanical stability of the spine [4, 9, 10]. Compared to open instrumentation, the minimally invasive treatment causes less tissue trauma as well as reduced blood loss and fewer wound healing disorders [4, 6, 11, 12]. Nonetheless, some studies suggest that with minimally invasive dorsal instrumentation techniques using polyaxial pedicle screws less restoration of the spinal alignment can be achieved with higher loss of reduction compared to open procedures [1, 7, 8, 13–19].

Particularly in the short-segment fixation of thoracolumbar vertebral body fractures, the increased mechanical stress on the polyaxial pedicle screws can lead to a reduction of the segmental lordosis [20, 21]. However, previous studies have shown that the patients’ clinical outcomes and the postoperative quality of life does not correlate with the radiographic outcomes [11, 22, 23].

Purpose of this study was to examine the restoration of the sagittal spinal alignment and the postoperative loss of reduction of surgically treated spinal fractures. Secondary goal of this study was to measure the extend of loss of reduction during the follow-up period and to analyze if a loss of reduction leads to less favorable clinical outcomes.

The authors hypothesize that polyaxial pedicle screws might not prevent a sagittal loss of reduction during fracture healing. However, we also hypothesize that this loss of reduction or kyphotic deformity will not lead to any functional impairment.

## Methods

### Study design and population

**Fig. 1:**
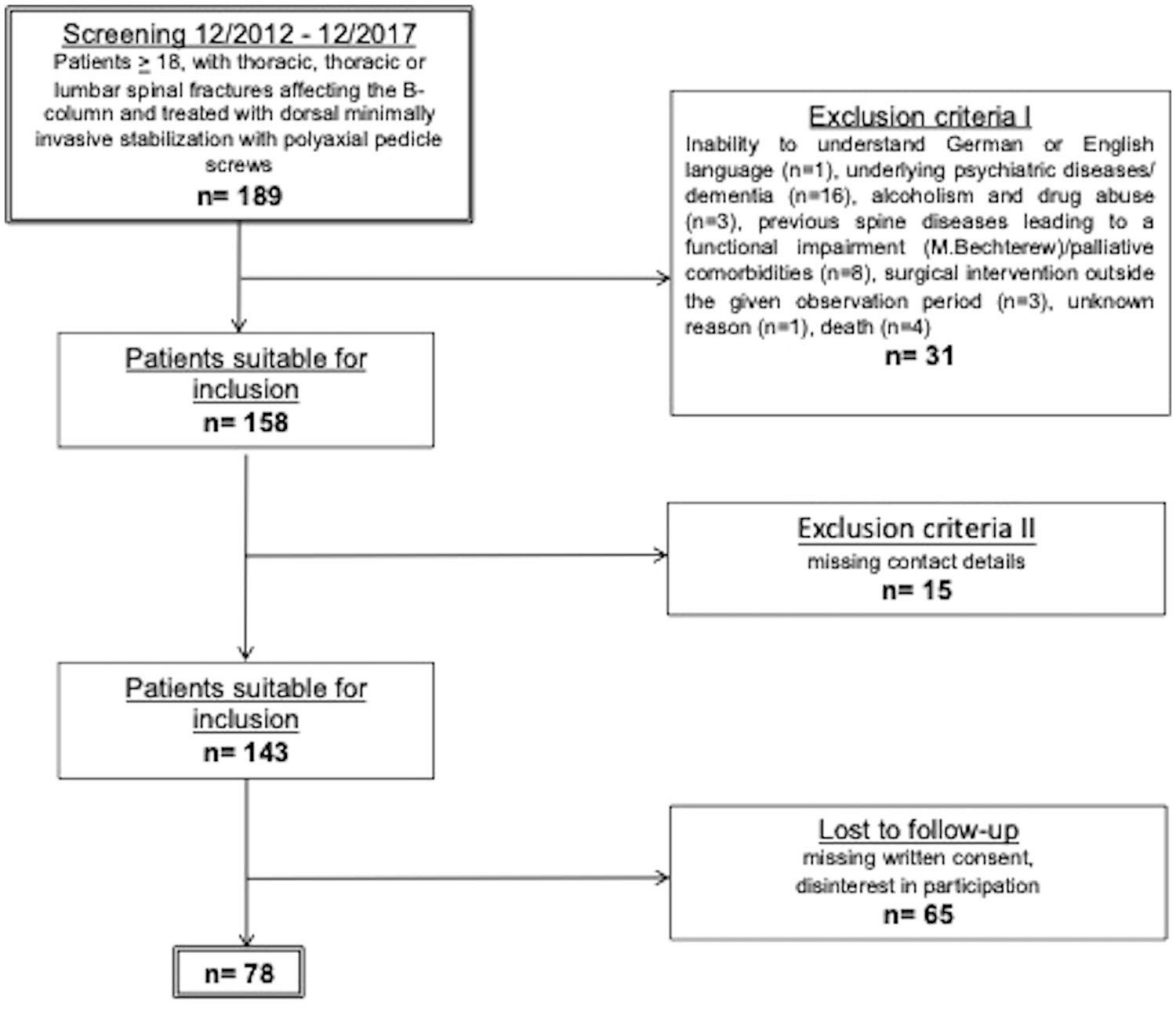
Patient collective with inclusion and exclusion criteria

This was a single-center study at a Level I Trauma Center including 78 patients, 40 (51.3%) men and 38 (48.7%) women, who underwent a dorsal minimally invasive stabilization of thoracic or lumbar spinal fractures, over a 5-year period (from December 2012 to December 2017). It is a prospective cohort analysis, according to a Level II c study. The study protocol was approved by the local ethics committee (No.: 5430), and all patients gave their informed written consent to participate in the study. All patients with a fracture of the spine from the 3rd thoracic to the 5th lumbar vertebral body who had undergone a percutaneous minimally invasive internal fixator with polyaxial pedicle screws were included, provided that they consented to participate in the study and in the follow-up. Patients that received a laminectomy were excluded. Further inclusion criteria comprised patients older ≥ 18 at the time of the accident and a written consent to participate in the study protocol. Patients with relevant pre-existing conditions of the spine such as previous fractures or deformities, rheumatoid arthritis or ankylosing spondylitis were excluded from the study (Fig. 1).

Indications for surgical treatment were based on the fracture type according to the AO Spine Classification. Here, all type A2 and A4 as well as type B and C fractures were treated surgically. Within the type A3 fractures, decision for surgical intervention was based on the following criteria: fracture-related kyphotic deformity (vertebral body angle ≥ 20°), relevant involvement of the posterior edges, and narrowing of the spinal canal.

According to the treatment protocol of our clinic, single vertebral body fractures were treated bisegmentally and multiple vertebral body fractures were treated multisegmentally, independent of the localization of the fracture. Open surgical procedures and laminectomies were excluded from the study.

### Operative procedure

All 78 patients were treated via a minimally invasive dorsal approach by inserting pedicle screws in the pedicles of the vertebral bodies directly above and below the fracture. Reduction of the fracture was achieved by a ventral sag and distraction of the fractured segment following the principle of an internal fixator. Two different internal fixator systems were used for surgical stabilization of the vertebral body fractures. The Revolve^©^System from Globus Medical^®^ (Audubon, Pennsylvania, USA) (*n* = 63) and the USS Universal Spine System^©^ from Synthes^®^ (Raynham, Massachusetts, USA) (*n* = 15). A total of 24 (30.8%) of the implanted internal fixator systems were additionally stabilized by cement augmentation of the pedicle screws. The decision to use cement augmentation was made based on the intraoperative bone quality. If the bone quality was rather osteoporotic, cement augmentation was used to overcome complications like screw loosening and further inconveniences. Usually, cement augmentation is considered in all patients over an age of 60 due to poor bone quality.

Bi-segmental stabilization of the fractured vertebral segment was performed in 66 patients, whereas 12 patients underwent multi-level fusion. Mono-segmental treatment was not performed in any of the cases. An additional staged ventral spondylodesis was performed in 16 patients (20.5%). In two other patients, a recommendation to perform a ventral spondylodesis was available at the time of data collection. However, this had not yet been performed.

As a result of this procedure, complications were documented in the postoperative course in 5 of the 16 patients operated on. One patient experienced an implant failure of the inserted material during the course of surgery, another patient experienced a collapse of the upper plate of the previously instrumented vertebral body, and three patients experienced postoperative complications not directly related to the surgical site.

### Data collection

Patients’ data were collected using electronic medical records. Descriptive base line patient data such as age, gender, relevant previous illnesses, the mechanism of injury and any accompanying injuries were recorded. Further, evaluation of the fracture localization, the fracture classification according to the AO Classification, the type of screw system used, the number of instrumented vertebral bodies, and all perioperative and postoperative complications were recorded.

### Measurement of vertebral body angle and mono-segmental/bi-segmental wedge angle

The vertebral body angle and the mono-segmental and bi-segmental wedge angle were measured using digitally stored radiographs, including X-ray, CT and / or MRT images. Radiological images were assessed using the SkyVue digital program, a picture archiving and communication system (Cerner^®^, Missouri, USA), and the containing measurement tools. Measurements were performed immediately preoperatively and postoperatively as well as after an average follow-up examination period of 8.5 (SD 8) months, with a median follow-up of 6.5 months. All measurements were carried out by the same examiner using an established and standardized protocol [24] and the results were reviewed by an experienced spine surgeon with at least 30 years of experience.

### VAS spine score

The subjective patient outcome, referring to pain, was measured using the VAS spine score, a standardized and validated questionnaire for the assessment of pain and well-being of patients suffering from thoracolumbar fractures. The VAS spine score is based on the VAS spinal scores of the German Society for Trauma Surgery [25]. The patient processed the questionnaire after an average of 18 ± 12 months (median: 13 months).

### Statistical analysis

For statistical analysis, the software programs Microsoft Excel^®^ 2016 (Version 1902, Microsoft, Redmond, WA, USA) as well as IBM SPSS^®^ (Version 23, IBM Inc., Armonk NY, USA) were used. The Kolmogorov–Smirnov and Shapiro–Wilk tests were used to test for normal distribution of the data. Since none of the data to be analyzed had a normal distribution, the comparison of means was performed using the following non-parametric tests for statistical significance: Mann–Whitney test, Friedman test, Wilcoxon signed-rank test, and Kruskal–Wallis test. *p* values ≤ 0.05 were considered statistically significant.

## Results

### Patient collective and fracture classification

In our collective of 78 patients, a total of 88 vertebral body fractures were diagnosed.

The average age at the time of the accident was 61 ± 17 years (median: 64 years) with a range of 18 to 86 years. Differentiated by gender, the mean age of men was 58 ± 17 years (median: 61 years) with a range of 24–82 years, and that of women was 64 ± 17 years (median: 68 years) with a range of 19–86 years. The breakdown of patients into age groups shows an increase in vertebral fractures with increasing age (Fig. S1). Only in the age group ≥ 80 years, the number of patients with a vertebral body fracture decreases again. Most patients with a vertebral body fracture were between 50 and 79 years of age (*n* = 47).

We mainly included patients, where the spine injury was the leading injury.

Concomitant injuries presented by patients that were included did not impair mobilization significantly.

Fifty-six patients suffered a mono-injury. One or more concomitant injuries were documented in 22 patients, 4 of whom met the criteria for polytrauma (Injury Severity Score ≥ 16). All polytrauma patients had a traffic accident. Because some patients sustained more than one concomitant injury, a total of 30 concomitant injuries were documented. Concomitant injuries were: an additional vertebral body fracture (12.8%), other vertebral body injury without neurological deficits (3.5%), other fracture, n.d. (5.8%), blunt thoracic trauma (4.7%), blunt abdominal trauma (3.5%), craniocerebral trauma (2.3%), intracranial hemorrhage (2.3%).

In 56 patients, the vertebral fracture was the only injury. One or more accompanying injuries were documented in 22 patients, 4 of which met the criteria of a polytrauma (Injury Severity Score ≥ 16). All polytrauma patients were involved in a traffic accident.

The fractures were localized between the 3rd thoracic vertebra and the 5th lumbar vertebra and classified according to their spinal region: thoracic spine (T1 to T10), thoracolumbar junction (T11 to L2), and lumbar spine (L3 to L5). There was a peak incidence of fractures in the area of the thoracolumbar junction (*n* = 56), the majority affecting L1 (*n* = 30), followed by T12 (*n* = 15) (Table 1).

**Table 1:** AO spine classification and fracture localization

Localization of fracture	AO spine classification							Total
	A1	A2	A3	A4	B1	B2	C	
Thoracic spine (T1–T10)	1	3	4	2	2	1	2	15
T3	0	0	1	0	0	0	0	
T4	0	0	0	0	1	0	0	
T7	0	0	1	1	0	0	0	
T8	0	0	1	1	0	1	1	
T9	0	2	1	0	0	0	1	
T10	1	1	0	0	1	0	0	
Thoracolumbar junction (T11–L2)	6	3	38	9	0	0	0	56
T11	1	0	2	0	0	0	0	
T12	2	1	10	2	0	0	0	
L1	3	2	18	7	0	0	0	
L2	0	0	8	0	0	0	0	
Lumbar spine (L3–L5)	2	1	10	2	1	1	0	17
L3	1	0	5	1	1	0	0	
L4	1	1	4	0	0	1	0	
L5	0	0	1	1	0	0	0	
Total	9	7	52	13	3	2	2	88

The severity of the fractures was assessed using the AO Spine classification [26]. The majority of the fractures (59.1%, *n* = 52) corresponded to the A3 fracture group (incomplete burst fracture), 14.8% of the fractures (*n* = 13) could be assigned to the group of A4 fractures (complete burst fracture), 10.2% (*n* = 9) to the group of A1 fractures (compression fracture), and 8.0% of fractures (*n* = 7) to the group of A2 fractures (split or pincer fracture). The remaining fractures were evenly distributed among B1, B2, and C fracture groups. Overall, it is noticeable that all fractures diagnosed at the thoracolumbar junction (T11 to L2) belonged to group A and all complex fracture types (group B and C) were located outside the thoracolumbar junction (Table 1).

### Complications and implant removal

Intraoperative complications occurred in 4 out of 78 patients (5.1%). Prolonged postoperative bleeding from the operation site was observed in one patient. Another patient presented a mal-positioning of a pedicle screw, which was corrected intraoperatively. Furthermore, cement extravasation was found in one patient and a failed cement augmentation of a pedicle screw was found in another. In the further postoperative course, 21 patients (26.92%) presented various complications, the majority of them being minor complications without indication for revision surgery. Major complications with indication for revision surgery showed overall a small incidence (Table 2). Secondary pedicle screw dislocations were observed in five patients, of which one patient showed neurological symptoms. In this case, the pedicel screws were cement augmented and an additional infection of the surgical site was reported. Within the other four patients with pedicle screw dislocation, without neurological symptoms, three received cement augmentation of the pedicle screws and one of them did not.

**Table 2:** Postoperative complications and their frequencies

Postoperative complications	Prevalence (*n*)	Prevalence (%)
No complications	57	73.1
Complications not related to operation area	5	6.4
Cement extravasation	4	5.1
Screw dislocation without neurological symptoms	4	5.1
Wound healing disorders	2	2.6
Subsidence upper end plate	2	2.6
Implant failure	2	2.6
Implant-associated infection	1	1.3
Screw dislocation with neurological symptoms	1	1.3
Total	78	100.0

Over the entire follow-up period, implant removal of the internal fixator was performed in 19 of the 78 patients (24.4%). Reasons for the implant removal were mainly patient-based requests after radiologically verified sufficient fracture consolidation (*n* = 12)

Fracture consolidation was verified depending on fracture type, classified by the official AO Spine Classification. Here, type A1 impression fractures were verified by conventional radiological imaging. Fracture healing in type A2 to A4 fractures was evaluated by native computer tomography.

Other reasons for implant removal were: undesirable effects such as screw loosening (*n* = 2), implant failure (*n* = 2), wound healing complications (*n* = 2) or deep surgical site infections (*n* = 1).

In our study, screw loosening was detected in two patients. In these cases, most likely, pre-existing osteoporosis was underestimated and cement augmentation was not performed. Implant failure was also seen in two cases, mainly in patients with complete burst fractures, where additional ventral surgery was not performed because of missing consent. We have included this information into our manuscript.

### Determination of kyphotic deformity and reduction results for all patients

The extent of the fracture-related kyphotic deformity was determined in a total of 76 patients on the basis of X-ray, CT or MRI images. For two patients (2.6%), no initial images were available to determine the fracture-related kyphotic malalignment. The vertebral body angle and the mono-segmental and bi-segmental wedge angles of the affected spine section were measured and recorded. The average preoperative, fracture-related kyphotic deformity measured for the vertebral body angle was 11.4° ± 5.5° (median: 10.5°, range: 2°–26°). For the mono-segmental wedge angle, we measured 13.0° ± 6.1° (median: 13.5°, range: 1°–26°) and for the bi-segmental wedge angle, we found 14.5° ± 9.9° (median: 11.5°, range: 1°–51°).

**Fig. 2:**
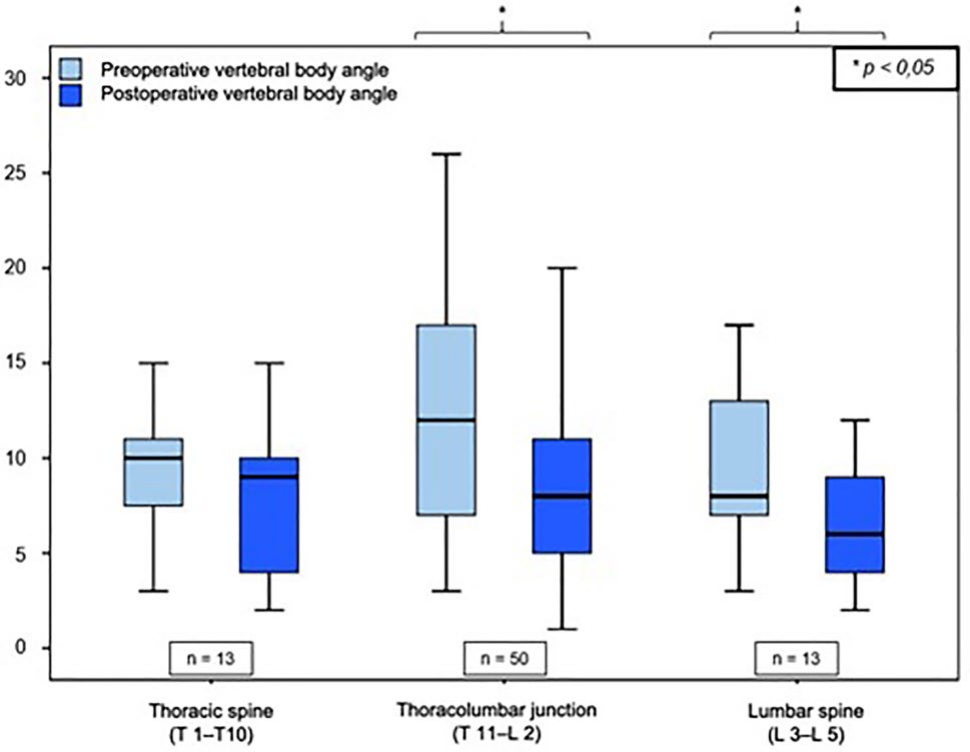
Comparison of vertebral body angle before and after surgical stabilization

**Fig. 3:**
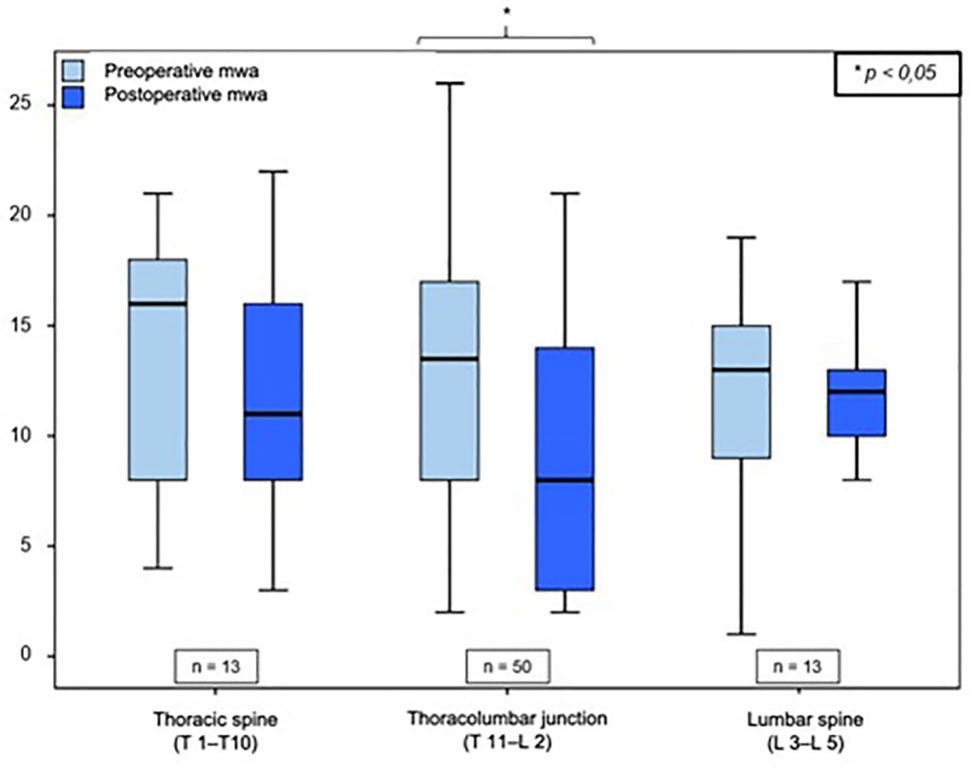
Comparison of mono-segmental wedge angle (mwa) before and after surgical stabilization

**Fig. 4:**
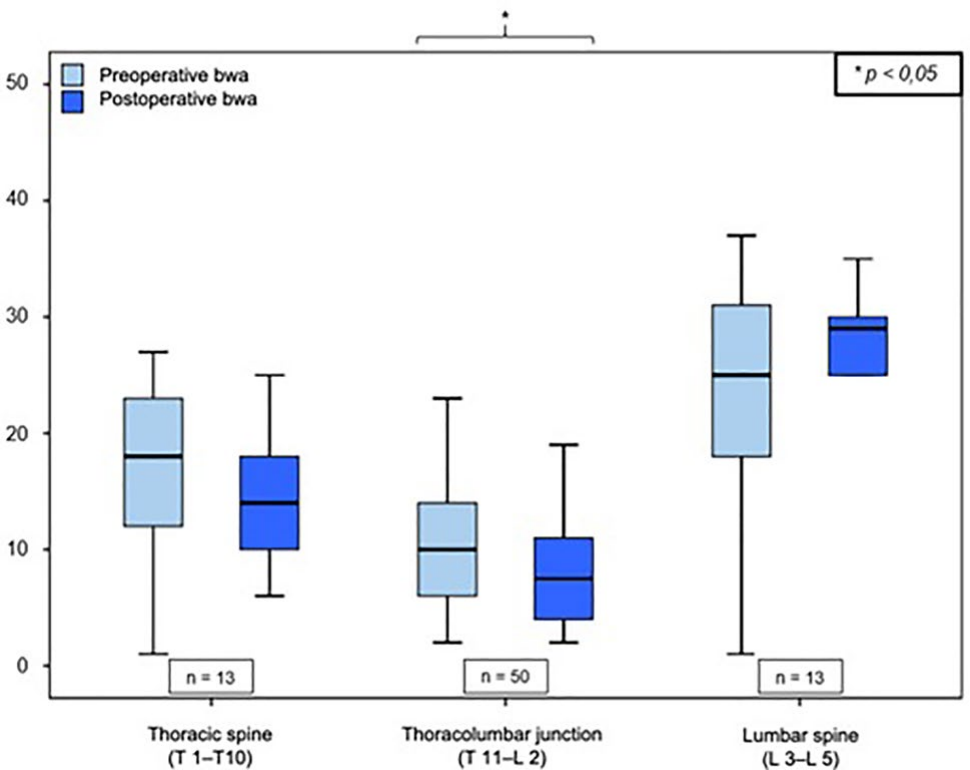
Comparison of bi-segmental wedge angle (bwa) before and after surgical stabilization

Immediately postoperatively, the remaining fracture-related kyphotic deformity of the vertebral body angle was 8.2° ± 4.7° (median: 8.0° range: 1–25°), which reports an improvement of 3.2° ± 4.4° compared to the preoperative value (11.4° ± 5, 5°; *p* = 0.000). The mean postoperative mono-segmental wedge angle was 9.9 ± 5.8° (median: 9.8°, range: 1°–23°), and thus the kyphotic deformity of the affected spine segment could be corrected by 3.1° ± 5.6° compared to the preoperative value measured (13.0° ± 6.1°; *p* = 0.000). The mean postoperative bi-segmental wedge angle was 12.5 ± 9.7° (median: 10.0°; range: 2°–47°). Therefore, the fracture-related kyphotic deformity improved by 2.0° ± 6.3° compared to the preoperative value (14.5 ± 9.9°, *p* = 0.281). These results demonstrate a significant improvement (Wilcoxon sign-rank test, *p* < 0.05) of the fracture-related kyphotic malalignment in relation to all three postoperatively determined angles. Comparison of preoperative and postoperative angles can be seen in Figs. 2, 3 and 4.

**Fig. 5:**
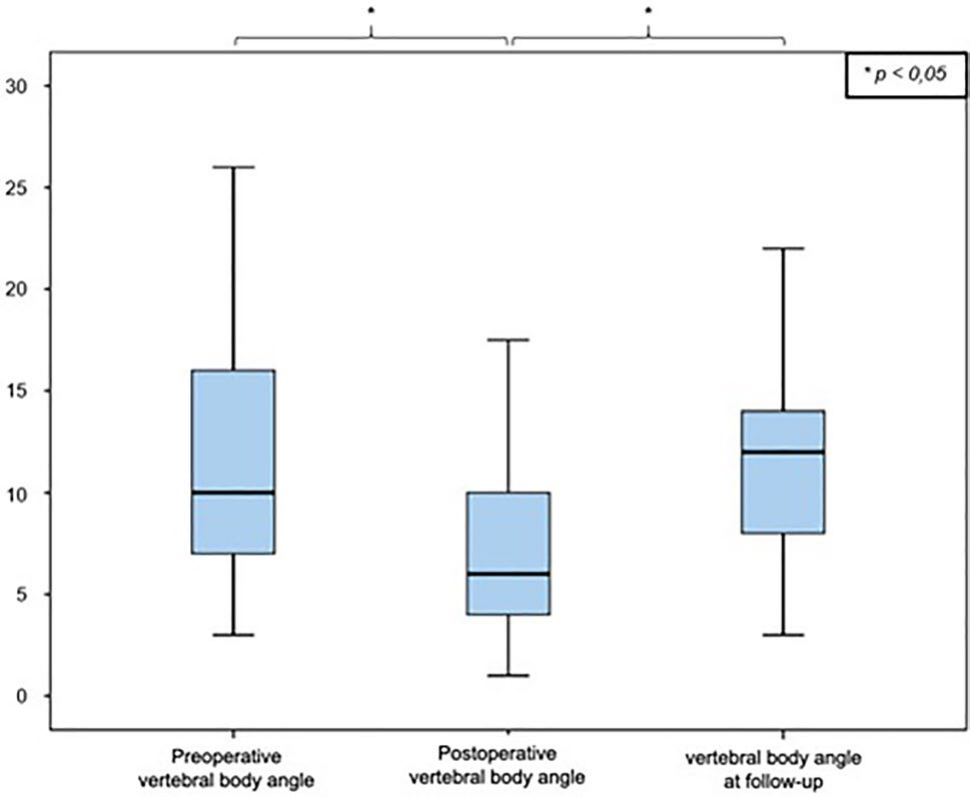
Development of the vertebral body angle preoperatively and postoperatively and at the time of follow-up

**Fig. 6:**
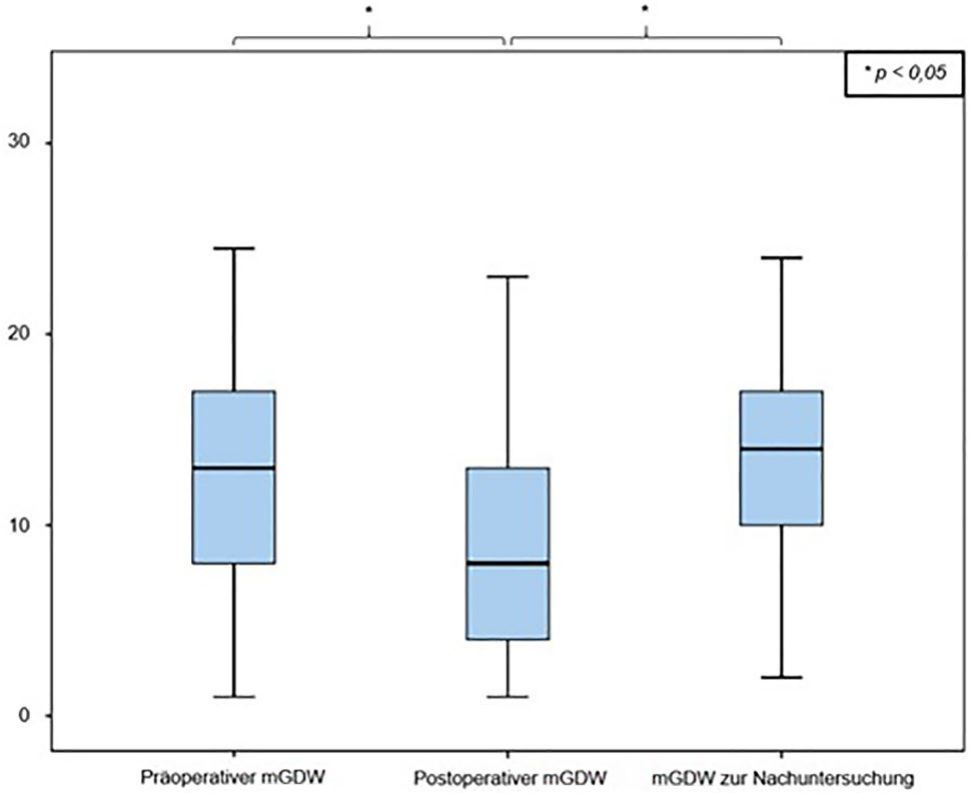
Development of the mono-segmental wedge angle (mwa) preoperatively and postoperatively and at the time of follow-up

**Fig. 7:**
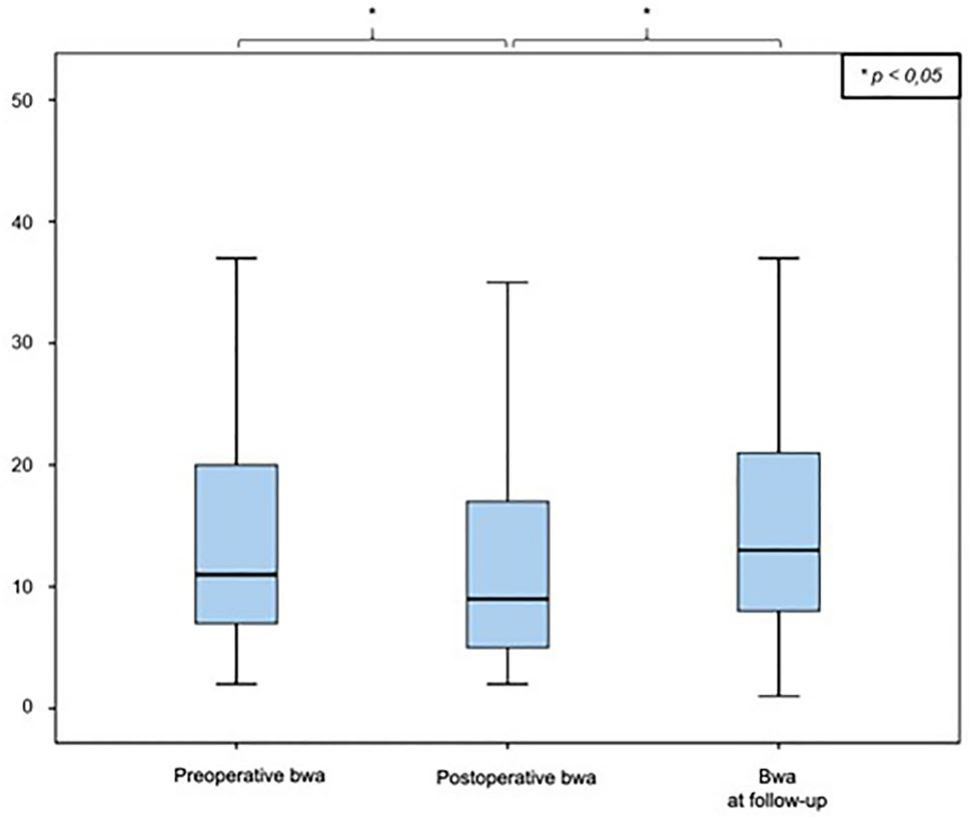
Development of the bi-segmental wedge angle preoperatively and postoperatively and at the time of follow-up

At the time of the follow-up examination, the vertebral body angle showed a mean value of 12.1° ± 5.9° (median: 12.0°, range: 3°–30°), indicating a significant loss of reduction of 4.5° ± 5.8° compared to the postoperatively measured body angle (Wilcoxon signed rank test, *p* < 0.000) (Fig. 1). The mean mono-segmental wedge angle was 13.8° ± 6.8° at the time of the follow-up examination (median: 14.0°, range: 2°–33°), showing a significant loss of reduction of 4.7° ± 6.3° since operative stabilization (Wilcoxon signed rank test, *p* < 0.000) (Fig. 2). The mean bi-segmental wedge angle at the time of follow-up was measured as 16.0° ± 11.0° (median: 13.0°, range 1°–45°), also showing a significantly loss of reduction of 3.7° ± 7.5° since operative treatment (Wilcoxon signed rank test, *p* < 0.001) (Figs. 5, 6, 7).

All three radiological parameters measured (vertebral body angle, mono-segmental and bi-segmental wedge angle) showed a consecutive, secondary loss of reduction over the postoperative course, which was significant at the end of the follow-up period compared to the immediately postoperative reduction results (Figs. 5, 6, 7).

Additionally, an analysis and comparison of all cement-augmented pedicel screws with non-cement-augmented pedicle screws showed no statistically significant differences with regard to all three time points (preoperative and postoperative follow-up) in all three radiological angles (vertebral body angle, mono-segmental wedge angle, bi-segmental wedge angle) (Table 3).

**Table 3:** Comparison of radiological angles between cement-augmented and non-cement-augmented pedicle screws

Variable	Cement-augmented	Non-cement-augmented	*p* value
Number of patients (*n*)	(26)	(60)	
Preoperative: mean (SD)			
Vertebral body angle	12.2 (6.0)	10.9 (5.1)	0.457
Mono-segmental wedge angle	14.1 (6.7)	12.3 (5.8)	0.314
Bi-segmental wedge angle	15.4 (10.9)	13.9 (9.2)	0.781
	(26)	(62)	
Postoperative: mean (SD)			
Vertebral body angle	9.5 (5.2)	8.0 (4.6)	0.217
Mono-segmental wedge angle	11.2 (6.9)	9.4 (5.1)	0.290
Bi-segmental wedge angle	13.1 (9.2)	12.4 (9.3)	0.670
	(15)	(45)	
Follow-up: mean (SD)			
Vertebral body angle	12.9 (7.1)	11.1 (5.4)	0.675
Mono-segmental wedge angle	17.4 (10.6)	12.2 (5.4)	0.060
Bi-segmental wedge angle	18.7 (15.2)	15.3 (8.9)	0.878

### VAS spine score

During follow-up, 76 patients completed the VAS spine score after an average time period of 18 ± 12 months (median: 13 months, range: 4–48 months). Across the patient cohort, a mean score of 65 ± 23 points (median: 72 points, range: 7–98 points) was obtained. There was no significant correlation between the VAS spine score result and the extent of fracture-related kyphotic deformity at the time of the accident (Pearson correlation coefficient: vertebral body angle: *p* = 0.06, mono-segmental wedge angle: *p* = 0.161, bi-segmental wedge angle: *p* = 0.135) nor between the VAS spine score result and secondary loss of reduction at the time of follow-up (Pearson correlation coefficient: vertebral body angle: *p* = 0.256, mono-segmental wedge angle: *p* = 0.286, bi-segmental wedge angle: *p* = 0.312).

## Discussion

Controversy exists regarding treatment options to restore stability in fractures of the thoracic and lumbar spine without neurologic disorder [1, 3, 10, 15, 17]. Stable fractures of the spine are treated conservatively [27, 28]. However, this can increase the risk of immobility especially in older patients with comorbidities [29]. Minimally invasive surgical treatment options are increasingly used for fractures without neurologic deficits and in older patients [4]. The present study analyzes the restoration of the sagittal spinal alignment of the fractured spine section after minimally invasive, dorsal stabilization using a polyaxial pedicle screw system. Moreover, the extend of the loss of reduction after a mean follow-up period of 8.5 months was evaluated. Immediately after surgical intervention, a significant improvement in the fracture-related sagittal profile could be observed. Regarding the analyzed patient’s subjective pain score, there was no correlation between the radiological result and patient satisfaction in terms of back pain. Similar results can be found in the literature [23, 30, 31]. Wang et al. [9] compared groups of patients that were stabilized with polyaxial pedicle screws in a minimally invasive procedure with a group of patients who were instrumented with monoaxial screws. In all groups, there was a significant improvement of the immediate fracture-related kyphotic malalignment through surgical therapy. A decent correction loss of 1.4° (vertebral body angle) and 3.6° (bi-segmental wedge angle) was observed in the follow-up period, that showed an average of 20 months, with quite a wide range from 12 to 48 months. Compared to the vertebral body angle, the reduction of the fracture-related kyphotic deformity after surgical therapy, and the correction loss in the follow-up period, the results of our study were less successful. In contrast, the extent of the accident-related kyphotic deformity as well as the correction loss measured using the bi-segmental wedge angle in the follow-up period were almost identical to our results. It should be mentioned that Wang et al. only investigated type A fractures, while our study collective also included a number of type B and C fractures, which have a higher fracture-related instability per se. Although an additional comparison of A1 to A3/4 fractured in our study only showed a significant difference regarding the preoperative mono-segmental wedge angle, but not in the further.

Spiegl et al. [32] found a significantly higher loss of reduction (10.8°, measured with the bi-segmental wedge angle) when using polyaxial implants compared to monoaxial implants (2.8°, measured by the bi-segmental wedge angle). In addition, better results in terms of postoperative reduction were recorded by inserting an index screw into the fractured vertebral body or by cement augmentation of the inserted pedicle screws.

The question whether monoaxial or polyaxial pedicle screws should be used in posterior stabilization of vertebra fractures remains a topic of discussion. However, currently there is no definitive guideline.

In general, polyaxial screws are prone to fatigue failure especially at the junction between the screw head and the shaft [33, 34]. In contrast, monoaxial screws with incorporated screw and shaft result in a generally stiffer construct [35].

Yao et al. published an article in 2021 where they compared the efficacy of monoaxial pedicle screws vs. polyaxial pedicle screws in short-segment posterior fixation for the treatment of thoracolumbar fractured vertebra. They found that compared with polyaxial pedicle screws, monoaxial pedicle screws endow stronger leverage which is more beneficial for restoring injured vertebral height and recovery of the damaged endplate in thoracolumbar short-segment posterior fixation [36].

Other studies that compared monoaxial vs. polyaxial screws in thoracolumbar fractures documented that monoaxial percutaneous pedicle screws inserted at adjacent fracture levels provided significantly better fracture reduction compared to polyaxial screws [37] and better radiological results [38].

Although monoaxial screws seem to have improved rotational control compared with polyaxial screws, their use may increase screw–bone interface or vertebral endplate forces if not inserted in an exactly straight trajectory [39], which in turn may lead to screw loosening [40], especially because monoaxial screws lead to a generally stiffer construct of the posterior stabilization.

In our study, we could not show significant differences with regard to reduction loss, between cement-augmented and non-cement-augmented pedicle screws. The decision of using cement augmentation was rather eminence based than evidence based, and took place intraoperatively by the surgeon.

The use of fenestrated screws allowing cement injection became an essential tool in patients with osteoporosis or reduced bone quality to limit the risk of fixation failure and allow a higher stability of the construct [41].

Since usually, a bone densitometry is not preoperatively routinely provided, especially not in patients with traumatic spine injuries, the use of cement augmentation in our patient collective was based on a clinical decision primarily considering the patients’ bone quality, injury pattern, and the patients’ age.

However, concerning polyaxial screw systems, also favorable study outcomes can be found in literature. Fitschen-Oestern et al. [7] compared minimally invasive stabilization of thoracolumbar vertebral body fractures using either a monoaxial or polyaxial pedicle screw systems with results of an open surgical technique. Postoperatively and after a 12-month follow-up period, no significant differences between these two procedures (monoaxial or open surgery) could be determined. A correction loss of 6.2° (bi-segmental wedge angle) was found when using polyaxial pedicle screws. Although, the determined correction loss of 6.2° is about twice as high, compared to the correction loss determined in our study (3.7°). Fitschen-Oestern et al. advocate the minimally invasive stabilization of thoracolumbar vertebral body fractures with polyaxial pedicle screws because of a generally lower risk of perioperative complications and a lack of significant differences regarding the correction loss between the three applied procedures [7].

Further, other surgical methods for the treatment of vertebral body fractures were described and compared in literature. The multicenter study II of Reinhold et al. [23] compared data from patients with vertebral body fractures treated either conservatively, by surgical stabilization or by kyphoplasty or vertebroplasty. In the group of surgically stabilized patients, depending on the fracture morphology, a posterior, anterior or a combined surgical approach was chosen. The majority of the posterior operations were performed with an angle-stable internal fixator system. The fracture-related degree of kyphotic deformity was, regardless of the procedure, corrected by 4.7° (bi-segmental wedge angle) on the lumbar spine (thoracic spine: 6.1°, thoracolumbar junction: 9.3°). After a follow-up period of 15 months, there was an average correction loss of 3.1° (bi-segmental wedge angle) at the lumbar spine. The loss of correction was more pronounced at the thoracic spine (4.6°) and at the thoracolumbar junction (4.8°). The greatest loss of correction was observed after a sole posterior stabilization. The fact that the results of the follow-up examination did not reveal whether they were recorded before or after an implant removal is a limitation of this study [23]. It can be assumed that the removal of the implant will result in subsequent subsidence of the previously fractured spinal column section [42]. A comparison of the rate of postoperative correction loss at the follow-up period from the study of Reinhold et al. [23] to our results showed a distinctly lower loss of reduction in our results, despite the use of a polyaxial fixator system and an overall implant removal rate of 25.6%. Additionally, the study by Reinhold et al. showed significantly poorer radiological results regarding the fracture-related kyphotic malalignment in conservatively treated patients compared to surgically treated patients.

Finally, the choice of fixation system is of substantial importance. Fusions with rigid fixation systems can lead to complications such as adjacent intervertebral disc degeneration, screw pullout or implant cutout [43], as mentioned before. These complications occurred very rarely in our study using polyaxial screws. Further, no statistically significant difference could be perceived with regard to loss of reduction or screw pullout in patients with cement-augmented systems and patients without.

With regard to loss of reduction, the bone quality of the patient is crucial. In our study, we included traumatic fractures of any quality of patients at any age that occurred after an adequate trauma and required surgical treatment. These patients possibly were accompanied by osteoporosis. We have not included any osteodensitometric measurements (typically not performed in patients with a fresh vertebral body fracture), and therefore any predictions with this regard are not valid and cannot be drawn from the results of our study.

Rigid fixations systems without additional ventral stabilization often lead to implant failure because of mechanical imbalance [44]. Therefore, a preventive implant removal is often necessary in those cases [45, 46].

In our study, the decision for an additional anterior stabilization was a two-staged decision, based on regular clinical and radiological follow-up examinations, where a ventral instability and/ or sintering of the vertebrae were seen, and therefore a stabilization was indicated and performed.

We usually did not perform an anterior stabilization in patients with A1 fractures, as it is usually not indicated and needed. Referring to the results, in a general sense, after an additional anterior stabilization, no more loss of reduction should occur and a secondary dislocation or failure of dorsal instrumentation is rather rare. Certainly, clinical results can differ and even show worse results compared to solely posterior stabilized patients, because of the higher grade of injury and the additional morbidity caused by an additional surgical intervention [47].

Our results show that implant failure was observed in only 2.6% (*n* = 2) of the patients, none of which having undergone a ventral stabilization. Implant removal was performed in 24.4% of the patients in our study. The most frequent reason was patients’ request after radiologically verified fracture consolidation. Less frequent reasons with medical indication were infections and the above mentioned two cases of implant failure. Thus, it has to be discussed, whether an implant removal is indicated, considering the impending loss of reduction in the affected motion segment. Here, the indication of implant removal should be precisely balanced in light of the patients’ wish.

Regardless of the reduction result and the extent of correction loss after surgical stabilization of the spine, the subjective clinical outcome of the patient should be regarded as the most important indicator for the success of the therapy and is, therefore, closely looked at by healthcare cost sponsors. In our study, the subjective treatment success of the patient was evaluated using the VAS spine score from Knop et al. [25]. No coherence was observed between subjective results of the VAS score and the radiological outcome in the follow-up period. Considering comparative literature, only one study supports a correlation between the extent of correction loss in the follow-up period and the results in the VAS spine score [48]. The majority of studies did not determine a causal correlation [23, 30, 31].

There are several limitations to the present study. First, there was a high number of patients (*n* = 65) that could not be included into the study due to disinterest in participation or missing written consent. Second, the interest in participating in the follow-up examinations of many patients was low, which explains our rather high number of loss of follow-up. We see reasons for this in the associated effort for the patients without a “recognizable”, personal benefit for them (e.g., financial compensation for participation), the relatively high age of the patients with the associated restriction of mobility and the effort of travel for those patients who did not live in the immediate vicinity of the hospital.

Retrospective studies in particular are usually associated with a high loss of follow-up. For this reason, we tried to restrict this loss of follow-up through a prospective approach by trying to recruit patients for the study during their inpatient stay. However, despite this, we could not achieve a higher study participation. Since many patients were asked about their participation in the study during their stay in hospital and not months later, we do not believe that there is a strong bias in our study, but of course this cannot be excluded.

Third, our study includes patients of different age groups which could lead to a bias of the results regarding bone quality, a loss of reduction over the follow-up. A higher age does not necessarily mean osteoporosis, but on the basis of degenerative and biological terms, a higher age is often accompanied with a reduced bone quality. Our results should be interpreted under awareness of these facts.

The follow-up period with an average of 8.5 months is relatively short and additionally states an overall wide time range. Moreover, successive subsidence of the bridged vertebral body could have been influenced by implant removal, performed in 19 of the 78 patients in our collective. However, as this was only performed in a total of 24% of the patients over the follow-up period, while a certain loss of reduction was found in 100% of cases, we did not assume a major implication here.

## Conclusion

Using a purely dorsal instrumentation with polyaxial pedicle screws, the sagittal alignment cannot be maintained permanently, despite optimal intraoperative reduction. This secondary loss of reduction as the main finding of this study could be due to micro-movements of the polyaxially directed screw heads under axial loading forces. In this case, the advantage of the minimally invasive implantation technique has to be put in contrast to the non-sustainable reduction result. However, the loss of reduction has no influence on the patients’ subjective outcome regarding pain. 

### Supplementary Information

Below is the link to the electronic supplementary material.

Number of vertebral body fractures broken down by sex and age groups (numbers in bars indicate the number, m = male, w = female) (JPG 29 KB)

## Data Availability

Research data associated with this paper is available on request to the corresponding author.
