# Supplementary figures and images for: Is restoration of vertebral body height after vertebral body fractures and minimally-invasive dorsal stabilization with polyaxial pedicle screws just an illusion?
Source: Arch Orthop Trauma Surg. 2023 Oct 15;144(1):239–50. doi: 10.1007/s00402-023-05082-8 (PMC10774198; doi:10.1007/s00402-023-05082-8)

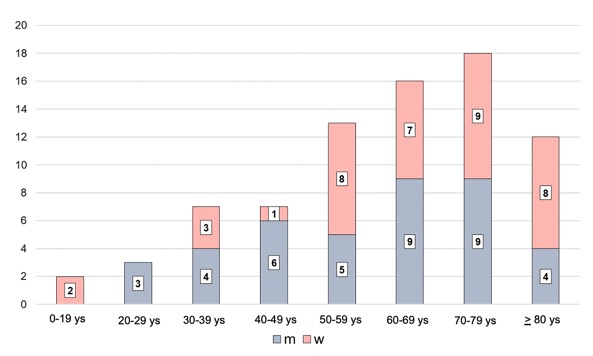

Supplement: Supplementary file 1 — Number of vertebral body fractures broken down by sex and age groups (numbers in bars indicate the number, m = male, w = female) (JPG 29 KB) [file 402_2023_5082_MOESM1_ESM.jpg]
